# Tissue-specific Co-expression of Long Non-coding and Coding RNAs Associated with Breast Cancer

**DOI:** 10.1038/srep32731

**Published:** 2016-09-06

**Authors:** Wenting Wu, Erin K. Wagner, Yangyang Hao, Xi Rao, Hongji Dai, Jiali Han, Jinhui Chen, Anna Maria V. Storniolo, Yunlong Liu, Chunyan He

**Affiliations:** 1 Department of Epidemiology, Richard M. Fairbanks School of Public Health, Indiana University, Indianapolis, IN, USA; 2 Department of Medical and Molecular Genetics, Indiana University School of Medicine, Indianapolis, IN, USA; 3 Department of Epidemiology and Biostatistics, Tianjin Medical University Cancer Hospital and Institute, National Clinical Research Center for Cancer, Tianjin & Key Laboratory of Cancer Prevention and Therapy, Tianjin, China; 4 Channing Division of Network Medicine, Department of Medicine, Brigham and Women's Hospital, and Harvard Medical School, Boston, MA, USA; 5 Indiana University Melvin and Bren Simon Cancer Center, Indianapolis, IN, USA; 6 Spinal Cord and Brain Injury Research Group, Department of Neurosurgery, Stark Neuroscience Research Institute, Indiana University, Indianapolis, IN, USA; 7 Susan G. Komen Tissue Bank at the Indiana University Melvin and Bren Simon Cancer Center, Indianapolis, IN, USA; 8 Center for Computational Biology and Bioinformatics, Indiana University, Indianapolis, IN 46202, USA

## Abstract

Inference of the biological roles of lncRNAs in breast cancer development remains a challenge. Here, we analyzed RNA-seq data in tumor and normal breast tissue samples from 18 breast cancer patients and 18 healthy controls and constructed a functional lncRNA-mRNA co-expression network. We revealed two distinctive co-expression patterns associated with breast cancer, reflecting different underlying regulatory mechanisms: (1) 516 pairs of lncRNA-mRNAs have differential co-expression pattern, in which the correlation between lncRNA and mRNA expression differs in tumor and normal breast tissue; (2) 291 pairs have dose-response co-expression pattern, in which the correlation is similar, but the expression level of lncRNA or mRNA differs in the two tissue types. We further validated our findings in TCGA dataset and annotated lncRNAs using TANRIC. One novel lncRNA, *AC145110.1* on 8p12, was found differentially co-expressed with 127 mRNAs (including *TOX4* and *MAEL*) in tumor and normal breast tissue and also highly correlated with breast cancer clinical outcomes. Functional enrichment and pathway analyses identified distinct biological functions for different patterns of co-expression regulations. Our data suggested that lncRNAs might be involved in breast tumorigenesis through the modulation of gene expression in multiple pathologic pathways.

Breast cancer is the most common invasive cancer and the leading cause of cancer death in women. It is a genetic disease of aberrant gene expression –the result of dysregulation of gene networks that maintain normal cellular functions and identity. In humans, only ~1.2% of the genome is protein-coding, and substantial fractions of the genome (~80%) can be transcribed into noncoding RNAs (ncRNAs) with no protein-coding capacity^1^. Recent research shows that ncRNAs, rather than transcriptional noise as previously believed, are capable of transacting a wide repertoire of regulatory functions^2^, suggesting the potential role of ncRNAs in shaping the genetic susceptibility of disease^3^.

Long noncoding RNAs (lncRNAs) are a class of ncRNAs with transcript size >200 nucleotides. They structurally resemble mRNAs but display a more tissue-specific expression pattern^4^. It is reported that lncRNAs might play widespread roles in gene regulation and other cellular processes, including acting as host genes for miRNAs, preventing miRNA, mRNA and proteins from binding with their intended targets, acting as molecular scaffolds, and serving as guides to direct proteins to their chromosomal targets^5^. Increasing evidence indicates that lncRNAs play an important role in cancer development and progression^6^. For example, the expression of HOTAIR is associated with metastasis and poor prognosis of breast cancer^7^. Despite the growing body of knowledge of lncRNAs, it remains a challenge to identify cancer-related lncRNAs on a genomic scale and further characterize their potential biological function in breast cancer development.

Co-expression analyses of protein-coding RNAs and lncRNAs have been reported to study the potential function of lncRNAs in biological processes and cancers^8^,^9^, including breast cancer^10^,^11^. However, these reports provide limited understanding of lncRNAs for the following reasons. First, most studies investigated co-expression of lncRNAs and mRNAs across a variety of tumor subtypes or states, but much less commonly between tumor and normal breast tissue. Second, in few studies, pathologically normal tissue adjacent to tumor, which was used as a baseline control, represented a suboptimal control as its expression profile has been shown to be altered in response to the adjacent tumor and was, to some extent, similar to that of tumor tissue^12^,^13^. Consequently, important breast cancer-associated lncRNAs may not be identifiable when using adjacent normal tissue as a baseline for comparison. Finally, most reports analyzed the co-expression of differentially expressed lncRNAs and mRNAs only, thus may fail to detect significant differences of the lncRNA-mRNA relationship between tumor and normal tissue but without dramatic changes of lncRNA or mRNA expression levels.

In this study, using RNA-sequencing data and normal breast tissue from healthy women as a desirable baseline control, we investigated the expression of lncRNAs in 18 breast cancer tumors and 18 normal tissue controls. Clinical information of the 18 breast cancer patients and the 18 healthy controls was summarized in Supplementary Table S1. We identified novel breast cancer-associated lncRNAs that were differentially expressed in breast tumor and normal tissue. Employing a genome-wide analytic approach, we further investigated co-expression of lncRNAs and mRNAs in breast tumor and normal tissue, and revealed two distinct co-expression patterns associated with breast cancer. Pathway analyses suggested different biological functions in tumorigenesis for each co-expression pattern. Overall, our study highlights the importance of lncRNAs in the carcinogenesis of breast cancer, and provides a valuable resource for lncRNA studies in cancer.

## Results

### Overview of the analysis pipeline

**Figure 1:**
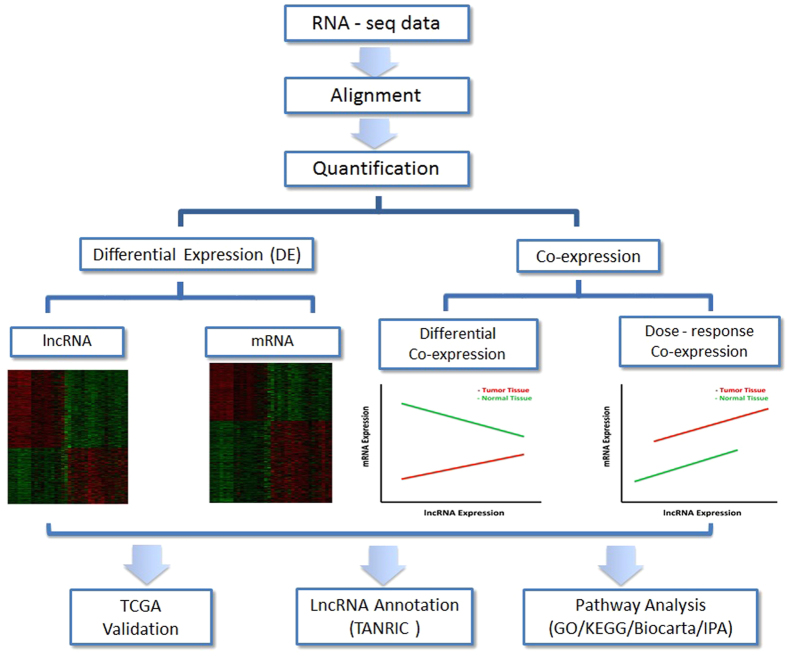
Flowchart of the analysis pipeline. We performed alignment and quantification on each RNA-seq sample, and then performed differential expression (DE) analysis to identify breast cancer-associated lncRNAs and mRNAs, as well as co-expression analysis between lncRNAs and mRNAs to infer potential function of lncRNAs considering two possible underlying mechanisms. Finally, our findings were validated using external TCGA dataset and other available bioinformatics resources including TANRIC functional annotation and pathway analysis.

The analysis pipeline is outlined in Fig. 1. We first performed differential expression (DE) analysis by comparing lncRNA or mRNA expression in tumor and normal breast tissue; we then analyzed co-expression of lncRNAs and mRNAs in tumor and normal breast tissue and revealed two distinct co-expression patterns associated with breast cancer; finally, we validated our findings using an external database, The Cancer Genome Atlas (TCGA) dataset, and further annotated lncRNAs using various bioinformatics resources and inferred their functional enrichment based on Gene Ontology (GO)/KEGG terms. Co-expression of lncRNAs and mRNAs in tumor and normal breast tissue was analyzed considering two scenarios: (1) differential co-expression in which the correlation between lncRNA and mRNA expression differs in tumor and normal breast tissue; and (2) dose-response co-expression in which the correlation is similar in tumor and normal breast tissue, but the expression level of lncRNA or mRNA differs in two tissue types.

### Differential expression of lncRNAs and mRNAs in tumor and normal breast tissue

We analyzed genome-wide expression of 7,450 lncRNA and 22,362 mRNA transcripts in tumor and normal breast tissue. We first investigated whether these transcripts could distinguish tumor from normal tissue. Unsupervised Principal Components Analysis (PCA) of lncRNAs demonstrated a clear separation of tumor from normal breast tissue (Supplementary Fig. S1a), similar to that of mRNAs (Supplementary Fig. S1b), illustrating the vast differences in their transcriptomic profiles. We also noted that lncRNAs distinguish tumor from normal tissue with three times fewer transcripts than mRNAs. This finding is in line with previous reports showing that lncRNAs display higher expression variation than mRNAs^14^.

**Figure 2:**
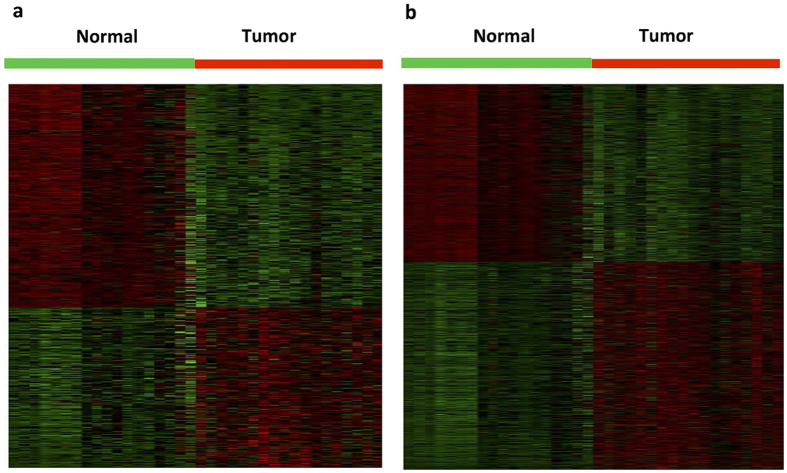
Expression differences of lncRNAs and mRNAs in breast tumor and normal breast tissue. Hierarchical clustering analysis of (**a**) differentially expressed lncRNAs; and (**b**) differentially expressed coding mRNAs between 18 breast tumor and 18 normal breast tissue samples (|fold change| ≥2 and FDR-adjusted *P* < 0.01). In the heatmap, columns represent samples, and rows represent each gene. Colors ranged from green (low expression) to red (high expression), represent the relative expression levels of lncRNAs and mRNAs.

We further performed a differential expression analysis to examine the differences in lncRNA and mRNA expression profiles between tumor and normal breast tissue. By the criteria of FDR adjusted *P* value < 0.01 and a two-fold change, we identified a total of 598 lncRNAs differentially expressed between tumor and normal breast tissue (Supplementary Fig. S2a; Supplementary Table S2). We found 348 lncRNAs (58.2%) down-regulated in tumor tissue (Fig. 2a). Similar reduction of expression levels have been reported for microRNAs in human cancer^15^, substantiating a common pattern of dysregulation of non-coding RNAs in carcinogenesis^3^. By the same criteria 2,980 mRNAs were identified to be differentially expressed between tumor and normal breast tissue (Supplementary Fig. S2b; Supplementary Table S3), consisting of 1,609 up-regulated (54.0%) and 1,371 down-regulated mRNAs (Fig. 2b).

The top ranked lncRNA in differential expression analysis, *RP11-118E18.2*, shows higher expression in breast tumor compared to normal tissue (FC = 16.7, FDR *P* = 5.49 × 10^−20^). Little is known regarding its biological function, but evidence from TANRIC^16^ shows differential expression of *RP11-118E18.2* between carriers and non-carriers of mutations in clinically actionable genes including *TP53*, *NAV3*, *MUC5B* and *MAP1A*. Additionally, *RP11-118E18.2* shows differential expression associated with ER status (*P* = 0.00026) and PR status (*P* = 0.001), PAM50 (*P* = 0.00046) and breast cancer therapy based on molecular signatures (*P* = 0.01) (Supplementary Fig. S3). However, the function remains largely unknown for most of the differentially expressed lncRNAs identified in our study, which is consistent with a previous report by Reiche *et al*.^10^. We also observed consistent direction of differential expression in tumor and normal tissue for majority of known cancer-related lncRNAs in two studies, though our study in general had larger fold changes than the study by Reiche *et al*.^10^ (Supplementary Table S4).

Enrichment analysis of differentially expressed mRNAs demonstrated that these genes are involved in cancer-related pathways, such as cell cycle, PPAR signaling pathway, apoptosis, and transcriptional dysregulation (Supplementary Fig. S4; Supplementary Table S5). We further employed IPA software to predict upstream transcriptional regulators that are either “inhibited” or “activated” based on the entire set of the 2,980 mRNAs that were differentially expressed in tumor and normal breast tissue^17^. The top list of regulator genes are enriched for cancer-related genes including *TGFB1* (*P* = 9.20 × 10^−29^)^18^, *TNF* (*P* = 1.43 ×  × 10^−21^)^19^, *TP53* (*P* = 1.83 × 10^−21^)^20^ and *ER* (*P* = 1.15 × 10^−18^)^21^, supporting the biological functions of the differentially expressed mRNAs relevant to breast cancer.

### Co-expression of lncRNAs and mRNAs in tumor and normal breast tissue

#### Differential co-expression analysis

We identified 516 lncRNA-mRNA pairs that were significantly and differentially co-expressed between tumor and normal breast tissue (Supplementary Table S6), of which 26 pairs (5.0%) were located on the same chromosome (*cis*-acting) and the remaining 490 pairs (95.0%) on different chromosomes (*trans*-acting), suggesting that most of the lncRNAs regulate mRNA expression in *trans*. These findings are in agreement with a recent study by Guttman *et al*., which reported that 92% of the lncRNAs were *trans*-acting in their study^22^. Details of regulation patterns for each chromosome are displayed in Supplementary Table S7.

Among those 516 lncRNA-mRNA pairs, we only detected 131 unique lncRNAs and 294 unique mRNAs. Seventy-five lncRNAs (57.3%) showed differential co-expression with just a single mRNA, while 56 (42.7%) showed differential co-expression with at least two mRNAs. On the other hand, 212 mRNAs (72.1%) showed differential co-expression with just a single lncRNA, while 82 (27.9%) showed differential co-expression with at least two lncRNAs. The top lncRNAs and mRNAs with the largest numbers of associations in differential co-expression are listed in Table 1.

Of interest, *AC145110.1* was found differentially co-expressed with 127 mRNAs in tumor and normal breast tissue, acting as a master regulator. Pathway analyses suggested these mRNAs are enriched in biological functions related to “cellular growth and proliferation”, “cell–to–cell signaling and interaction”, and “Hematological System development and function” (Supplementary Fig. S5). TANRIC analysis shows the lncRNA *AC145110.1* is correlated with disease stage of breast cancer (*P* = 0.0029), ER status (*P* = 0.0159), and HER2 status (*P* = 0.019) in the TCGA samples (Supplementary Fig. S6). Additionally, the TCGA samples show differential expression of *AC145110.1* between carriers and non-carriers of mutations in 19 clinically actionable genes. Taken together, these results suggest that *AC145110.*1 may play an important role in cancer development through long-range regulation of expression for multiple cancer-related genes.

### Dose-response co-expression analysis

Using the criteria described in the methods, we identified a total of 291 lncRNA-mRNA pairs that were dose-response co-expressed between tumor and normal breast tissue (Supplementary Table S8), of which 115 pairs (39.5%) located on the same chromosome and 176 pairs (60.5%) on different chromosomes, representing *cis*- and *trans*- acting regulation, respectively. Similar to the differential co-expression results, these findings are consistent with the recent findings that lncRNAs mostly regulate mRNA expression in *trans*^22^. Details of regulation patterns for each chromosome are displayed in Supplementary Table S9.

Those 291 lncRNA-mRNA pairs are represented by 149 unique lncRNAs and 262 unique mRNAs. Of which, 111 lncRNAs (74.5%) showed dose-response co-expression with a single mRNA, while the remaining 38 (25.5%) with at least two mRNAs. On the other hand, 235 mRNAs (89.7%) showed dose-response co-expression with just a single lncRNA, while 27 mRNAs (10.3%) with at least two lncRNAs. The top lncRNAs and mRNAs with the largest numbers of associations in dose-response co-expression are listed in Table 1.

### Validation in TCGA dataset

#### Differentially expressed mRNAs

**Figure 3:**
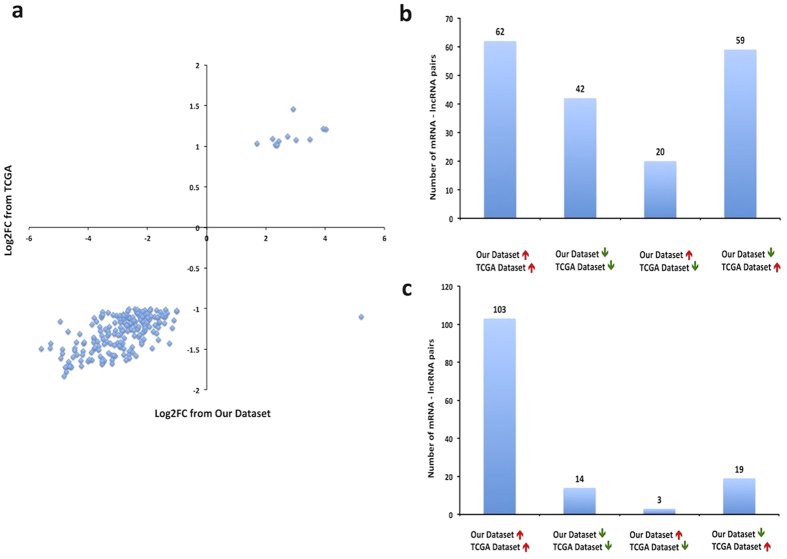
Validation in TCGA. (**a**) Validation of mRNA expression differences in TCGA. X-axis represents Log_2_FC between 18 breast tumor and 18 normal tissue samples from our dataset; Y-axis represents Log_2_FC between 744 breast tumors and 104 adjacent normal tissue samples from TCGA dataset; 207 differentially expressed mRNAs in both datasets are shown. (**b**) and (**c**) Validation of lncRNA-mRNA co-expression in breast tumors in TCGA. Our dataset consists of 18 breast tumor samples; and TCGA dataset consists of 692 breast tumors. (**b**) Directions of the lncRNA-mRNA associations in two datasets were compared in 183 lncRNA-mRNA pairs identified in differential co-expression analysis; (**c**) Directions of the lncRNA-mRNA associations were compared in 139 lncRNA-mRNA pairs identified in dose-response co-expression analysis. Red arrows indicate positive correlations, and Green arrows indicate negative correlation between lncRNAs and mRNAs.

We validated the differentially expressed mRNAs identified in our study using TCGA dataset of 848 breast tissue samples, consisting of 744 breast tumors and 104 adjacent normal breast tissue samples from women of European ancestry. Out of 14,371 mRNAs we analyzed in TCGA dataset, we identified 263 mRNAs differentially expressed between TCGA tumor and adjacent normal breast tissue with FDR adjusted *P* values < 0.01 and a two-fold change. Of these, 207 were also differentially expressed in our data. We found 99.5% of these mRNAs showed consistent direction of differential expression across two datasets (Fig. 3a). Of note, the fold change for a specific mRNA was generally larger in our dataset than in TCGA.

#### Co-expression of lncRNAs and mRNAs in breast tumors

We further validated the co-expression of lncRNAs and mRNAs in 692 breast tumor samples from the TCGA dataset. After quality control, only 6,556 lncRNAs and 15,074 mRNAs were retained for co-expression analysis in TCGA tumor tissue samples. Of the 516 differentially co-expressed lncRNA-mRNA pairs identified in our data, only 183 pairs were available to analyze in TCGA data, and 56.8% of these overlapping pairs had consistent direction of co-expression correlations in TCGA and our datasets (Fig. 3b). Of the 291 lncRNA-mRNA pairs we identified in our dose-response co-expression analysis, we were only able to analyze 139 pairs in TCGA data, and 84.2% of these overlapping pairs had consistent direction of co-expression correlations in TCGA and our datasets (Fig. 3c). These results may reflect larger variability in differential co-expression, when compared to dose-response co-expression between tumor and normal breast tissue. While high concordance rate for dose-response co-expression attested the validity of our data, the lower concordance rate for differential co-expression might be due to the different distribution of tumor characteristics (e.g. subtypes) in our dataset and TCGA dataset, as it is conceivable that differential co-expression is likely more sensitive to changes in tumor characteristics.

### Functional characterization of the identified lncRNAs

To better understand the function of the lncRNAs identified in our dataset, we employed an analytic pipeline to assess two aspects of the identified lncRNAs: (1) annotation of their co-expressed mRNAs; (2) their co-localization with known breast cancer risk loci.

**Figure 4:**
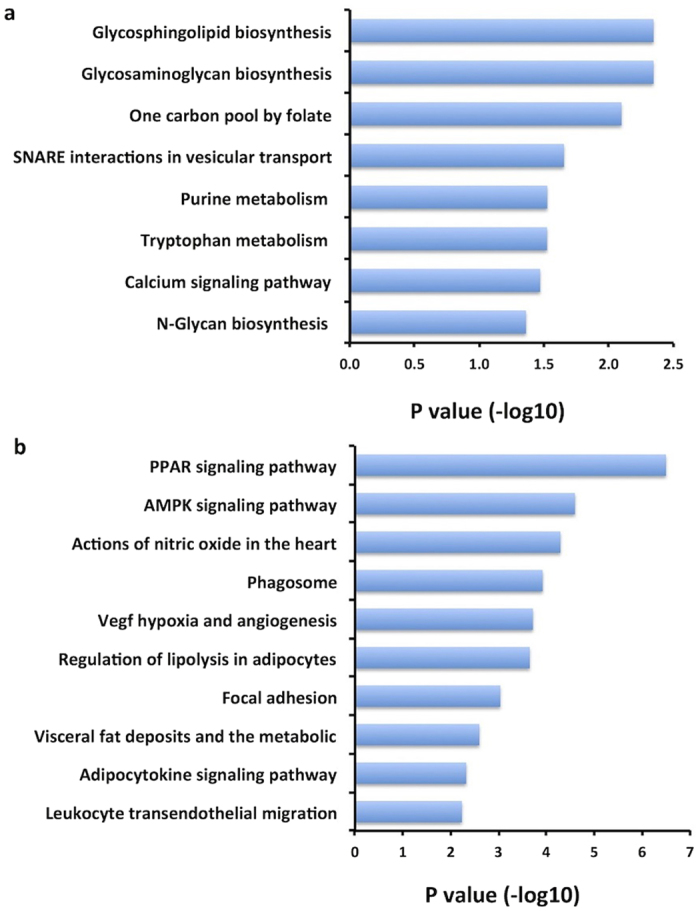
Functional enrichment for the mRNAs identified from lncRNA-mRNA co-expression analysis in our data. **(a**) mRNAs co-expressed with lncRNAs in differential co-expression network; (**b**) mRNAs co-expressed with lncRNAs in dose-response co-expression network.

To infer biological function of lncRNAs, their co-expressed mRNAs were subjected to Gene Ontology (GO), KEGG, and BioCarta annotations. The 294 mRNAs showing differential co-expression with lncRNAs were enriched for functions including “Glycosaminoglycan biosynthesis”, “Glycosphingolipid biosynthesis”, and “One carbon pool by folate” (Fig. 4a). It is intriguing to note the third function is methylation-related, suggesting that epigenetic mechanism might play an important role in lncRNA-mRNA differential co-expression regulation between tumor and normal breast tissue. Interrogation of 262 mRNAs that were co-expressed with lncRNAs in dose-response fashion reveals cancer-related pathways including “PPAR signaling pathway”, “AMPK signaling pathway”, “VEGF hypoxia and angiogenesis”, and “adipocytokine signaling pathway” (Fig. 4b). GO analysis showed similar results for functional enrichment (Supplementary Table S10).

Secondly, we investigated co-localization of lncRNAs with known breast cancer risk loci. We identified 368 SNPs from the NHGRI-EBI Catalog of published GWAS of breast cancer^23^ (Nov, 2015 accessed). The majority of these genetic loci fall into non-coding regions^24^. Mapping these loci to the genomic regions, we found 44 loci located within 43 lncRNA regions (Supplementary Table S11). Of those, three risk loci (rs9832625, rs11836164 and rs2823779) are located in lncRNAs that were identified in either differential expression or co-expression analysis in our study (Table 2). Of note, lncRNA *LINC00478* harbors SNP rs2823779, a risk locus associated with toxicity after radiotherapy in breast cancer patients^25^. This lncRNA was found differentially co-expressed with four mRNAs in our study, including *TOX4* and *CETN1*.

### Cross Validation

#### Overlap between the identified lncRNAs

We compared the lncRNAs identified in differential expression, differential and dose-response co-expression, and assessed the overlap specific to each comparison (Supplementary Fig. S7). We observed 119 lncRNAs overlapping in dose-response co-expression and differential expression, which was as expected due to our significance criteria used for dose-response co-expression. Interestingly, few lncRNAs overlap between differential and dose-response co-expression, indicating that most lncRNAs might exclusively involve in distinct co-expression regulations with different underlying mechanisms. We also observed the small overlap of lncRNAs between differential expression and differential co-expression. Intriguingly, a significant proportion of lncRNAs identified in differential co-expression were not differentially expressed between tumor and normal breast tissue. This may be due to the very stringent *P* value threshold we used in differential co-expression. It is also possible that lncRNAs regulate mRNA expression differently in tumor and normal tissue but without significant changes of their own expression levels.

#### Overlap with conventional co-expression analysis

Previous studies commonly used a conventional approach that only analyzed the co-expression of lncRNAs and mRNAs that are both differentially expressed between tumor and normal tissue^26^,^27^. We employed a genome-wide approach to analyze co-expression of lncRNAs and mRNAs in tumor and normal breast tissue and applied a stringent significance threshold to guard against false positives. Out of the 516 lncRNA-mRNA pairs identified in our differential co-expression, only 8 pairs had both differentially expressed lncRNAs and mRNAs (Table 3). The overlap was more pronounced in dose-response co-expression analysis. Out of the 291 pairs identified in our dose-response co-expression, 162 pairs had both differentially expressed lncRNAs and mRNAs (Supplementary Table S12). These results suggested that our approach might be more robust to identify co-expression, especially differential co-expression pattern, between lncRNAs and mRNAs that were associated with breast cancer.

#### Co-expression patterns of known breast cancer-related lncRNAs

We further investigated known breast cancer-related lncRNAs in our study. We found *HOTAIR* and *HOTAIRM1* were differently expressed between tumor and normal breast tissue based on our criteria (Supplementary Table S13). This finding is consistent with previous research^28^. For co-expression analysis, only two of these lncRNAs, *MALAT1* and *XIST*, were found differentially co-expressed with three mRNAs (*TOX4, ALG14*, and *C12orf32*) in our data (Table 4). No known breast cancer-related lncRNAs were found significant in our dose-response co-expression analysis.

## Discussion

In past decades, transcriptomic studies have focused on the analysis of protein-coding transcripts to characterize their patterns and potential functional roles. The recent development of next-generation sequencing technology has greatly accelerated the discovery and characterization of a new class of non-coding RNA transcripts, lncRNAs. Increasing evidence suggests that lncRNAs have key regulatory functions in chromatin remodeling and gene expression in the progress of disease, including cancer^3^. Indeed, a few dysregulated lncRNAs, such as *HOTAIR* and *DSCAM-AS1*, have been linked to breast cancer^29^. However, the role of lncRNAs in breast cancer development remains largely unknown. Using high-throughput RNA sequencing technology and a computational approach, we systematically evaluated genome-wide expression of lncRNAs and co-expression between lncRNAs and mRNAs in tumor and normal breast tissue. Our study identified novel breast cancer-associated lncRNAs and inferred their potential biological and pathological roles in gene regulation and cancer development.

Although emerging evidence indicates that lncRNAs play a key role in many biological processes such as cell differentiation, immune response, and tumorigenesis, the functions of lncRNAs are not well understood^30^. In order to functionally characterize lncRNAs, a “guilt by association” strategy is commonly used to construct a co-expression network of lncRNAs and mRNAs^31^. In the conventional approach, differential expression analysis is first performed in tumor and normal tissue and only the identified differentially expressed lncRNAs and mRNAs are analyzed in the following co-expression network^11^,^26^. While this approach significantly reduces the number of tests, it might fail to detect some important, cancer-related co-expression relationship between lncRNAs and mRNAs that shows no significant change in expression level of either RNA. In this study, we employed a genome-wide approach and systematically investigated lncRNA-mRNA co-expression in a serial of statistical models using stringent significance threshold. We considered two possible co-expression patterns related to breast cancer, that is, differential and dose-response co-expression networks. Indeed, compared to the conventional approach, our genome-wide approach identified significantly more lncRNA-mRNA co-expression relationship associated with breast cancer. Of particular interest, the conventional approach only identified less than 2% of differential co-expression pairs in our genome-wide approach, while this proportion is 55% for dose-response co-expression pairs. These results suggested our genome-wide approach is more robust to identify breast cancer-associated lncRNA-mRNA co-expression, especially for differential co-expression network.

Most previous studies investigated transcriptome profiling in tumor and adjacent normal tissue from cancer patients, in which histologically normal tissue adjacent to the tumor was commonly used as a baseline control because of its ready availability. However, adjacent normal tissue represents a suboptimal control, because its molecular profile has been shown to be altered in response to the adjacent tumor and is, to some extent, similar to that of tumor tissue^13^. Consequently, lncRNA expression or lncRNA-mRNA co-expression pattern is more similar or having smaller difference in tumor and adjacent normal tissue when compared to that in tumor and normal tissue from healthy controls. As a result, important breast cancer-associated lncRNAs or lncRNA-mRNA co-expression network may not be identifiable when using adjacent normal tissue as a baseline for comparison. Furthermore, comparing tumor tissue from cancer patients to normal breast tissue from healthy women will allow the identification of novel breast cancer- associated lncRNAs and lncRNA-mRNA co-expression that are influenced by individuals’ different genetic background and environmental risk factors, reflecting individuals’ susceptibility to the disease that is also biologically important to breast cancer development. This subset of molecular changes is unidentifiable when comparing tumor to adjacent non-tumorous or contralateral normal breast tissue from the same patients, because the germline genetic variation and environmental exposures are identical in those matched types of tissue. Indeed, we not only identified more significant findings but also observed the fold change for a specific transcript was generally larger between tumor and normal tissue in our dataset than that between tumor and adjacent normal tissue in TCGA. To our knowledge, our study is the first one that used normal breast tissue from healthy women as a more desirable baseline to identify breast cancer-related lncRNAs and lncRNA-mRNA co-expression network on a genome-wide scale. Our findings were not only consistent with previous studies on several known breast cancer-related lncRNAs and mRNAs, such as *HOTAIR*^7^, *BRCA2*^32^, *MMP9* and *MMP11*^33^, but more importantly, our study was able to identify novel breast cancer-related lncRNAs and lncRNA-mRNA co-expression networks. For examples, we observed a novel lncRNA, *RP11-118E18.2*, was significantly over-expressed in breast tumors (FC = 17, FDR *P* = 5.49 × 10^−20^). Although its biological mechanism has not been elucidated, validation by TANRIC confirmed its associations with multiple breast cancer clinical outcomes. We also found that two lncRNAs, *CAHM* and *KCNQ1DN*, are down-regulated in breast tumors compared to healthy normal controls in our study. These two lncRNAs has been found down-regulated in colorectal cancer^34^ and glioblastoma tumors^35^ but no previous studies linked them to breast cancer. For breast cancer-associated lncRNA-mRNA co-expression pattern, we found lncRNA *AC145110.1* was differentially co-expressed with multiple mRNAs in tumor and normal breast tissue, including *CETN1* and *MAEL*. CETN1 is a cancer testis antigen that is highly expressed in prostate and pancreatic cancer^36^. Knockdown of CETN1 inhibits breast cancer cell proliferation^37^. *MAEL* is also a cancer testis gene regulated by DNA methylation. It interacts with stress gene in cancer cells and promotes hepatocellular carcinoma metastasis by inducing epithelial-mesenchymal transition^38^. This implies lncRNA *AC145110.1* is likely to be a hub of the lncRNA-mRNA regulatory network and may thus be involved in the important process of breast cancer development.

Our study revealed two lncRNA-mRNA co-expression patterns associated with breast cancer, suggesting distinct underlying regulation mechanisms. It is noteworthy that there is little overlap of lncRNAs involved in differential and dose-response co-expression networks, indicating most lncRNAs were exclusively involved in one of the two co-expression regulations. GO and pathway analyses of genes involved in differential lncRNA-mRNA co-expression identified biological functions enriched in metabolic processes including folate metabolism; while genes involved in dose-response co-expression were enriched in signal transduction pathways. Although both co-expression regulation patterns show functional enrichments and pathways implicated in breast tumorigenesis, it is conceivable that differential co-expression might be more functionally “disruptive” compared to dose-response co-expression as the former leads to different lncRNA-mRNA correlations in breast tumor and normal tissue. The lncRNA-mRNA correlation is similar in tumor and normal breast tissue in dose-response co-expression, suggesting similar regulatory mechanism in two tissue types. As mRNA expression changes in response to lncRNA expression level in a dose-response fashion, we could speculate that the continuing changes in lncRNA or mRNA expression level would gradually introduce changes in molecular phenotypes and eventually lead to breast cancer, resulting in the observed differences in lncRNA or mRNA expression in tumor and normal breast tissue. Moreover, in both differential and dose-response co-expression analyses, we noticed the existence of the phenomenon that one lncRNA was co-expressed with multiple mRNAs as well as multiple lncRNAs were co-expressed with one mRNA (Table 1). This phenomenon appears more pronounced in differential co-expression than in dose-response co-expression, indicating a more complex regulatory relationship between lncRNAs and mRNAs in differential co-expression network.

Regulatory mechanisms underlying the co-expression of lncRNAs and mRNAs may involve competing endogenous RNA (ceRNA), transcription factors, DNA methylation, and copy number variation^39^. In our study, we observed the enrichment of DNA methylation-related pathways in differential co-expression, suggesting that epigenetics might be an important regulatory mechanism underlying the lncRNA-mRNA relationship for breast carcinogenesis. Recent studies showed that the majority of lncRNAs in human are produced from divergently transcribed protein-coding genes and that the divergent lncRNA/mRNA pairs exhibit coordinated changes in transcription^40^,^41^, representing *cis*-acting co-expression of lncRNAs and neighboring mRNAs. LncRNAs may also show a functional role in gene expression by targeting distant (*trans*-acting) coding genes^42^,^43^. While both *cis*- and *trans*-acting co-expression of lncRNAs and mRNAs were identified in association with breast cancer in our study, the proportion of *trans*-acting co-expression was significantly higher in differential co-expression than in dose-response co-expression network (95% vs. 60%), suggesting more complex, long-range mechanisms involved in differential co-expression regulation.

Given the importance of lncRNAs in biology and disease, there is great interest in defining functions of lncRNAs previously discovered. Our study provided a functional characterization of known breast cancer-related lncRNAs through lncRNA-mRNA co-expression. We found two breast cancer-related lncRNAs, *MALAT1* and *XIST*, were significantly and differentially co-expressed with mRNAs (*ALG14*, *TOX4*, and *C12orf32*) in tumor and normal breast tissue via *trans*-acting mechanism. *MALAT1* has been shown to be differentially expressed in multiple tumors, including breast cancer, prostate cancer, and lung cancer^44^. It was differentially co-expressed with *TOX4* and *ALG4* in our study. *TOX4* plays an important role in DNA damage response and cell cycle^45^, functions implicated in tumorigenesis. It is epigenetically regulated in breast cancer^46^. Of note, *TOX4* is the top mRNA with the largest number of associations in differential co-expression analysis, suggesting it is regulated by multiple lncRNAs. The other mRNA, *ALG14*, may involve in glycosylation and lipid metabolism^47^, but its role in carcinogenesis remains unknown. *Xist* has been identified as an important mediator of X inactivation^48^. It was differentially co-expressed with *C12orf32* (also known as *RHNO1*) in our study. Consistent with a previous report^49^, *C12orf32* found overexpressed in tumors in our data. This gene may also involve in the DNA-damage response^50^ and has been suggested as a novel anticancer molecular drug target (e.g. siRNA drugs)^49^. Our study provides a new source of functional annotating and prioritizing lncRNAs with potentially functional importance for downstream experimental validation.

The majority of breast cancer genetic susceptibility loci identified from genome-wide association studies (GWAS) fall into non-coding regions^24^. One post-GWAS challenge is to functionally characterize these genetic loci in the development of breast cancer. It is possible that genetic variants mapping to lncRNAs could play an important role in regulating gene expression levels via lncRNA-mRNA co-expression network. Our study found three breast cancer risk loci (rs9832625, rs11836164 and rs2823779) mapped to lncRNAs that were either differentially expressed or co-expressed with mRNAs in tumor and normal breast tissue. Of note, rs2823779 mapped to lncRNA *LINC00478*. The latter was differentially co-expressed with four mRNAs in the lncRNA-mRNA regulatory network, including cancer-related genes *CETN1*^36^,^37^ and *TOX4*^46^. In addition, intron of *LINC00478* encodes a miRNA cluster comprising *let-7c*, *miR-99a*, and *miR-125b* that might regulate HER2 signaling in breast cancer progression^51^. Our study attested the important roles of those mRNAs and lncRNAs in breast tumorigenesis.

We acknowledge a number of limitations in our study. First, the study power was limited due to the moderate sample size, especially for lncRNA-mRNA co-expression analyses. However, we took multiple testing into consideration and applied a stringent significance threshold to guard against false positive results. Furthermore, we validated our findings using external resources, including TCGA dataset and other public bioinformatics resources. Future studies with larger sample sizes are needed to validate the reported findings. Second, we employed a computational and bioinformatics approach to infer lncRNA functions. We integrated expression profiles of mRNAs and lncRNAs into co-expression models to study lncRNA characteristics in tumor and normal breast tissue. Although we identified a set of mRNAs that were co-expressed with lncRNAs, the detailed mechanism of gene regulation remains unknown. Experimental validation of lncRNA roles in “wet” lab is warranted. Third, we considered all breast cancer cases as one group in our study due to limited sample size. However, it is possible that breast cancer-associated lncRNAs and lncRNA-mRNA co-expression regulation are specific to histological and molecular subtypes. Subgroup analyses by breast cancer subtypes are warranted in future studies. Finally, we only considered one possible mechanism for the function of lncRNAs in gene regulation, that is, through correlations of gene expression levels between lncRNAs and mRNAs. LncRNAs can work through multiple other mechanisms such as chromatin remodeling, promoter demethylation, microRNA silencing, and acting as molecular scaffolds^5^,^42^,^43^. Thus future research is needed to take into account these possible mechanisms for a more complete understanding of lncRNA functions.

In conclusion, using normal breast tissue as a desirable baseline control and a genome-wide analytic approach, we identified a number of tissue-specific lncRNAs associated with breast cancer and further inferred their biological functional through lncRNA-mRNA co-expression analyses. Our findings suggest a complex and extensive role of lncRNAs in breast cancer development through regulating gene expression. Further work is needed to validate our findings and to understand the detailed molecular mechanism of specific lncRNAs implicated in breast cancer.

## Methods

### Study subjects and breast tissue samples

The study includes 18 breast cancer cases and 18 healthy controls of women of European Ancestry between ages 32 to 80 (Supplementary Table 1). Cases are patients with pathologically confirmed primary breast cancer diagnosed at one of three hospitals in Indianapolis, Indiana, between 1998 and 2009: Indiana University (IU) Hospital, Eskenazi Hospital (previously known as Wishard Hospital), and IU Simon Cancer Center (IUSCC). Controls are randomly selected from a pool of healthy women who donated both blood and normal breast tissue samples to the Susan G. Komen Tissue Bank at IUSCC between 2005 and 2009, and were free of breast cancer up to the time of donation. The participants completed a questionnaire on medical histories and health-related exposures at the time of donation. Controls are matched to cases based on ancestry and age (within 2 years). Breast tissue (untreated tumor or normal) biospecimens were collected from each case and control under standard operating procedures at IUSCC. All breast tissue samples were snap-frozen immediately after removal and stored in liquid nitrogen until processed, and were determined to be of high quality through histological and molecular quality control tests. Tumor samples were pathologically verified for high tumor content. Information concerning demographics, clinical data, and personal risk factors, including age, race, reproductive history, family history of breast cancer, are either extracted from medical records (for cases) or obtained through the questionnaires (for controls). Signed informed consent was obtained from each case or control prior to tissue samples collection. The study was approved by Indiana University institutional review board. The study was carried out in accordance with the approved guidelines.

### RNA-sequencing data

Total RNA was extracted from freshly frozen breast tissue (tumor or normal) samples using the Qiagen miRNeasy Mini Kit. Construction of cDNA libraries and subsequent RNA sequencing of paired-end reads (2 × 50 nt reads) were performed according to the standard Illumina protocol using the HiSeq2000 sequencing systems. The raw sequencing output was 25–35 million reads per sample. Quality control (QC) filtering was first performed on raw RNA-seq data to remove adapter sequences and poor quality bases using the FastqMCF clipping algorithm^52^. RNA-seq reads were then mapped by Bowtie v1.0.0^53^ to GENCODE lncRNA reference (release 17) and UCSC GRCh37/hg19 knownGene reference, respectively, for lncRNA and mRNA annotations. Transcript abundances were quantified using NGSUtils^54^. Samples were further filtered based on percentage of genes detected (less than 50%) and percentage of reads mapped to the reference (less than 25%). Extreme outliers were further identified and filtered from the dataset using principal component analysis (PCA). Low expression lncRNAs were filtered from the dataset based on counts per million (CPM) threshold of 1. After these steps, a total of 7,450 lncRNAs and 22,362 mRNAs were retained and used in further analyses.

### Statistical Analysis

All data analyses were performed using R and Bioconductor, unless otherwise noted. Details of analysis methods are described as follows.

#### Differential Expression

Differential expression (DE) analyses were performed using edgeR v2.6.12 implemented in the Bioconductor package^55^ to identify differentially expressed mRNAs or lncRNAs between tumor and normal breast tissue. Biological coefficients of variation between the samples were estimated using an empirical Bayes approach under the assumption that the data follows a negative binomial distribution. Differential expression between tumor and normal breast tissue was analyzed using a generalized linear model to regress RNA (lncRNA or mRNA) expression on tissue type, adjusting for age and sequencing batch. We referred to it as Model 1: Y_RNA_ = β_0 _+ β_1_X_Tissue Type _+ β_2_X_Batch _+ β_3_X_Age_. The false discovery rate (FDR) by Benjamini and Hochberg (BH) procedure^56^ was applied to correct for multiple testing. Statistical significance was defined as FDR *P* value <0.01 and a two-fold change (FC) of expression level between comparison of tumor and normal breast tissue. The heat map and locus-by-locus volcano plot were performed using R package.

#### Co-expression Analysis

Co-expression of lncRNAs and mRNAs in tumor and normal breast tissue was analyzed using a generalized linear model to regress mRNA expression on lncRNA expression, adjusting for age and sequencing batch. In our study, we were specifically interested in breast cancer-associated co-expression patterns that differ in tumor and normal breast tissue. We considered two scenarios: (1) differential co-expression in which the correlation between lncRNA and mRNA expression differs in tumor and normal breast tissue; and (2) dose-response co-expression in which the correlation is similar in tumor and normal breast tissue, but the expression level of lncRNA or mRNA differs in two tissue types. Accordingly, we constructed two generalized linear models to analyze the data:

Model 2: Y_mRNA _= β_0 _+ β_1_X_lncRNA _+ β_2_X_Tissue Type _+ β_3_X_lncRNA_·X_Tissue Type _+ β_4_X_Batch _+ β_5_X_Age_;

Model 3: Y_mRNA _= β_0 _+ β_1_X_lncRNA _+ β_2_X_Tissue Type _+ β_3_X_Batch _+ β_4_X_Age_.

A lncRNA is considered to be differentially co-expressed with a mRNA in tumor and normal breast tissue if its correlation differs in the two tissue types. In order to reduce false positives, Bonferroni correction was applied to control for multiple testing and a stringent P value threshold (P < 3 × 10^−10^ for the interaction term in Model 2) was applied to declare statistical significance. Statistically significant differential co-expression between a lncRNA and a mRNA was defined as *P* value for interaction term β_3_ < 3 × 10^−10^ from model 2. A lncRNA is considered to be dose-response co-expressed with a mRNA if its correlation is similar in tumor and normal breast tissue but the expression level of either the lncRNA or the mRNA differs in the two tissue types. Statistically significant dose-response co-expression was defined when all the following criteria are met: |β_3_| < 0.01 from model 2, *P* value for β_1_ < 3 × 10^−10^ from model 3, and either lncRNA or mRNA is significantly and differentially expressed in model 1.

#### Validation in TCGA Dataset

(1) Differentially expressed mRNAsRNA-seq data from 848 individuals was downloaded from TCGA, including 744 breast tumors and 104 non-tumorous adjacent-normal breast tissue samples. All samples were collected from individuals who self-reported as women of European ancestry. This dataset consisted of called gene counts for 20,531 mRNAs. We filtered out low expression transcripts based on percentage of samples (less than 50%) and CPM cutoff of 1. A total of 14,371 mRNAs were remained after filtering and used in the differential expression analysis by edgeR. The false discovery rate (FDR) method by the Benjamini and Hochberg (BH) procedure^56^ was applied to correct for multiple testing. Statistical significance was defined as FDR *P* value <0.01 and a two-fold change of expression level between comparison of tumor and adjacent normal breast tissue. (2) Co-expression patternsBeing approved, raw RNA-seq data and the corresponding clinical data (Biotab format) for 692 breast invasive carcinoma (BRCA) patients was acquired from TCGA. We chose patient barcodes as unique identifiers to build the connection of transcriptome data with clinical information. Sequencing alignment, expression qualification, and QC filtering were performed as previously described for our data. Finally, a total of 6,556 lncRNAs and 15,074 mRNAs were retained for co-expression analysis in tumor tissue from TCGA. We used a generalized linear model to regress mRNA expression on lncRNA expression, adjusting for age and sequencing batch. Bonferroni correction was used to control for multiple testing.

#### Functional Validation using Bioinformatics Resources

We used an open-access resource, TANRIC^16^, to interactively explore biological and clinical function of the lncRNAs in breast cancer based on TCGA dataset of 837 breast tumor samples and 105 adjacent normal samples. Analysis of variance (ANOVA) was used to examine if a lncRNA was differentially expressed across tumor subtypes or tumor stages. Student t-test was used to assess statistical significance of the difference in lncRNA expression between mutated and wild-type of a particular gene. To identify biological pathways that are significantly enriched among the differentially expressed mRNAs and the mRNAs that are co-expressed with lncRNA in tumor and normal breast tissue, we performed a hypergeometric test using consensusPathDB^57^ to calculate the enrichment significance based on annotation files from GO^58^, KEGG^59^, and BioCarta (www.biocarta.com). Functional enrichment analysis was also performed using Ingenuity Pathway Analysis (IPA) software (www.ingenuity.com). We also identified the top list of transcriptional regulators that explain the observed differential gene expression using Upstream Regulator Analytic tool^17^ implemented in IPA software. We further investigated whether the identified lncRNAs from our data contained any known breast cancer risk loci identified from previous genome-wide association studies (GWAS). GWAS Catalog^23^ was used to retrieve breast cancer-associated single nucleotide polymorphisms (SNPs).

## Additional Information

**How to cite this article**: Wu, W. *et al*. Tissue-specific Co-expression of Long Non-coding and Coding RNAs Associated with Breast Cancer. *Sci. Rep.* **6**, 32731; doi: 10.1038/srep32731 (2016).

## Supplementary Material

Supplementary Information

Supplementary Tables

## Figures and Tables

**Table 1:** Top lncRNAs and mRNAs with the largest numbers of associations in differential and dose-response co-expression analysis.

	lncRNA	lncRNA Chromosome	#mRNAs	mRNA	mRNA Chromosome	# lncRNAs
Differential co-expression	AC145110.1	8	127	TOX4	14	25
	RP11-136K7.2	5	36	CETN1	X	19
	TINCR	19	26	KIN	10	15
	RP11-680F20.12	11	18	CETN2	X	11
	CTB-131K11.1	17	17	TMEM41A	3	9
Dose-response co-expression	RP11-161M6.2	16	17	SLC19A3	2	4
	ADIPOQ-AS1	3	11	ACSM5	16	2
	CTD-2363C16.1	5	11	AIFM2	10	2
	CTD-2541J13.2	18	11	AK021888	5	2
	LINC00341	14	10	ANKRD20A2	2	2

**Table 2:** Significant differential expression and co-expression results for lncRNAs co-localized with known breast cancer risk SNPs in our data.

SNP	Position	Near Gene(s)	LncRNA	lncRNA-DE analysis		mRNA	Dose-response Co-expression analysis		Differential Co-expression analysis		
				Log2 Fold Change	FDR P-value		*β*^a^	*P* value b	*β*_ Tumor^c^	*β*_ Normal^c^	Interaction *P* value^d^
rs9832625	3p24.1	RBMS3	ENSG00000235904.1|RBMS3-AS3	−2.66	2.61E-04	—	—	—	—	—	—
rs11836164	12p12.1	SSPN - ITPR2	ENSG00000255750.1|RP11-283G6.5	−1.2	1.53E-03	—	—	—	—	—	—
rs2823779	21q21.1	LINC00478, MIR99AHG	ENSG00000215386.6|LINC00478	−1.69	2.88E-04						
						TOX4	—	—	1.48	4.26	2.73E-11
						CETN1	—	—	−0.08	−1.82	8.67E-11
						KIAA1253	—	—	5.16	3.02	1.25E-10
						CETN2	—	—	0.98	3.19	2.80E-10

^a^β refers to the change of mRNA expression level corresponding to each unit increase of lncRNA expression level in tumor and normal breast tissue, which was estimated from generalized linear model 3.

^b^*P* value was estimated from generalized linear model 3.

^c^β refers to the change of mRNA expression level corresponding to each unit increase of lncRNA expression level in tumor and normal breast tissue, respectively, which was estimated from generalized linear model 2.

^d^*P* value for interaction term (lncRNA· tissue type) was estimated from generalized linear model 2.

**Table 3:** Differential co-expression of lncRNAs and mRNAs by conventional approach.

lncRNA ID	lncRNA Name	lncRNA Chromosome	mRNA ID	mRNA Chromosome	*β*_ Tumor^a^	*β*_ Normal^a^	log_2_FC (lncRNA) b	log_2_FC (mRNA) b
ENSG00000263069.1	CTD-2047H16.4	17	CETN2	X	9.60	5.06	1.01	1.11
ENSG00000215386.6	LINC00478	21	CETN2	X	0.98	3.19	−1.69	1.11
ENSG00000267078.1	RP11-666A8.9	17	EPPK1	8	−0.63	2.99	1.37	2.09
ENSG00000253187.2	HOXA-AS4	7	FAM89A	1	−4.36	−2.48	−2.32	−2.89
ENSG00000229970.2	AC007128.1	7	KIAA1724	2	−3.47	−5.69	3.09	1.07
ENSG00000261716.1	RP11-196G18.22	1	RP11-426E5.2	10	−0.06	−7.32	1.22	−4.90
ENSG00000261716.1	RP11-196G18.22	1	ST6GALNAC3	1	0.43	−4.40	1.22	−1.91
ENSG00000253187.2	HOXA-AS4	7	ST6GALNAC6	9	−0.38	1.84	−2.32	−1.04

^a^β refers to the change of mRNA expression level corresponding to each unit increase of lncRNA expression level in tumor and normal breast tissue, respectively, which was estimated from generalized linear model 2.

^b^FC refers to the fold change of expression level in breast tumor versus normal tissue for mRNAs and lncRNAs, respectively.

**Table 4:** Significant results for known breast cancer-related lncRNAs in our data.

lncRNA ID	lncRNA/Reference	Position	lncRNA-DE analysis		mRNA-DE Analysis				Dose-response Co-expression Analysis		Differential Co-expression Analysis		
			Log_2_FC a	*FDR adjusted P* value	mRNA	position	Log_2_FC^a^	*FDR adjusted P* value	*β*^b^^,^^c^	*P* value^b^^,^^d^	β in Tumor^b^^,^^e^	β in Normal^b^^,^^e^	*P* value Interaction^b^^,^^f^
ENSG00000251562.3	MALAT1^29^	11	0.70	3.80E-02	ALG14	1p21.3	−0.12	8.18E-01	—	—	**1.24**	**−0.65**	**2.86E-10**
					TOX4	14q11.2	0.39	4.60E-01	—	—	**−4.05**	**−7.27**	**1.46E-10**
ENSG00000229807.5	XIST^60^	Xq13.2	−0.04	8.90E-01	C12orf32	**12p13.33**	**1.5**	**1.88E-05**	—	—	**2.30**	**−0.16**	**1.29E-10**
ENSG00000228630.1	HOTAIR^29^	**12q13.13**	**3.64**	**4.02E-08**	—	—	—	—	—	—	—	—	—
ENSG00000233429.5	HOTAIRM1^60^	**7p15.2**	**−1.19**	**5.64E-06**	—	—	—	—	—	—	—	—	—

^a^FC refers to the fold change of expression level in breast tumor versus normal tissue for mRNAs and lncRNAs, respectively.

^b^“-” refers to non-significant in our study.

^c^β refers to the change of mRNA expression level corresponding to each unit increase of lncRNA expression level in tumor and normal breast tissue, which was estimated from generalized linear model 3.

^d^*P* value was estimated from generalized linear model 3.

^e^β refers to the change of mRNA expression level corresponding to each unit increase of lncRNA expression level in tumor and normal breast tissue, respectively, which was estimated from generalized linear model 2.

^f^*P* value for interaction term (lncRNA· tissue type) was estimated from generalized linear model 2.
