# Supplementary material for: Tissue-specific Co-expression of Long Non-coding and Coding RNAs Associated with Breast Cancer
Source: Sci Rep. 2016 Sep 6;6:32731. doi: 10.1038/srep32731 (PMC5011741; doi:10.1038/srep32731)
Supplement: Supplementary Information [file srep32731-s1.pdf]

Tissue-specific Co-expression of Long Non-coding and Coding RNAs Associated with Breast Cancer

Wenting Wu[1*], Erin K.Wagner[1*], Yangyang Hao[2], Xi Rao[2], Hongji Dai[1, 3], Jiali Han[1, 4, 5], Jinhui Chen[6], Anna Maria V. Storniolo[7], Yunlong Liu[2, 8], Chunyan He[1, 4, 8]

Supplementary Notes

Supplementary Figures Legends

Supplementary Figure S1. Unsupervised principal components analysis (PCA) of expressed transcripts in tumors and normal breast tissue. Tumor from breast cancer patients was indicated in red and normal breast tissue from healthy women was indicated in green. Scatter plots of principal components (PCs) for a) lncRNAs; and b) mRNAs; respectively.

Supplementary Figure S2. **Volcano plots of the differential expression analysis in tumor and normal breast tissue.** a) lncRNAs; and b) mRNAs. Vertical dotted lines denote fold change = ±2 horizontal dotted line denote the FDR *P*- value = 0.01.

Supplementary Figure S3. Summary results for lncRNA *RP11-118E18.2* from TCGA BRCA dataset in TANRIC. a) Correlation analysis of the lncRNA expression level and ER status; b) correlation analysis of the lncRNA expression level and PR status; c) correlation analysis of the lncRNA expression level and PAM50; d) correlation

analysis of the lncRNA expression level and breast cancer therapy based molecular signatures; e) correlation analysis of the lncRNA expression level and *P53* mutation status.

Supplementary Figure S4: Functional enrichment for the mRNAs identified from differential expression analysis in our data.

Supplementary Figure S5: **Ingenuity pathway analysis (IPA) of mRNAs that were differentially co-expressed with lncRNA *AC145110.1*.** Green color represents down-regulated genes in breast tumor tissue, red color represents up-regulated genes in breast tumor tissue in our study.

Supplementary Figure S6: **Summary results for lncRNA *AC145110.1* from TCGA BRCA dataset in TANRIC.** a) Correlation analysis of the lncRNA expression level and disease stage; b) correlation analysis of the lncRNA expression level and ER status; c) correlation analysis of the lncRNA expression level and HER2 status; d) correlation analysis of the lncRNA expression level and PAM50; e) correlation analysis of the lncRNA expression level and breast cancer therapy based molecular signatures; f) correlation analysis of the lncRNA expression level and *XIRP2* mutation status.

Supplementary Figure S7: Venn Diagram of lncRNAs overlaps among differential expression, differential co-expression, and dose-response co-expression analyses.

Supplementary Tables (please refer to the separate excel documents).

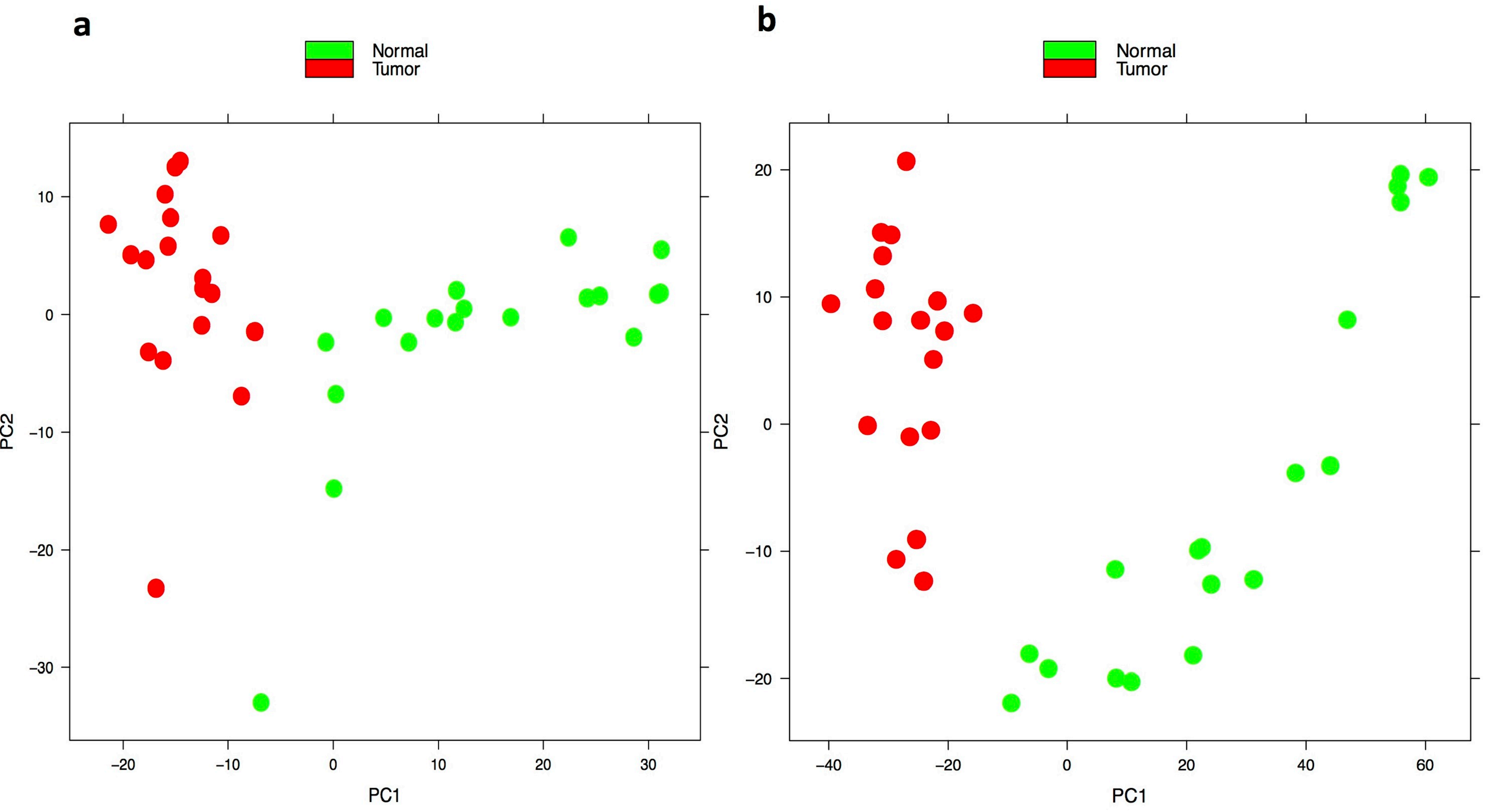

a
Normal
Tumor
PC1
PC2
b
Normal
Tumor
PC1
PC2

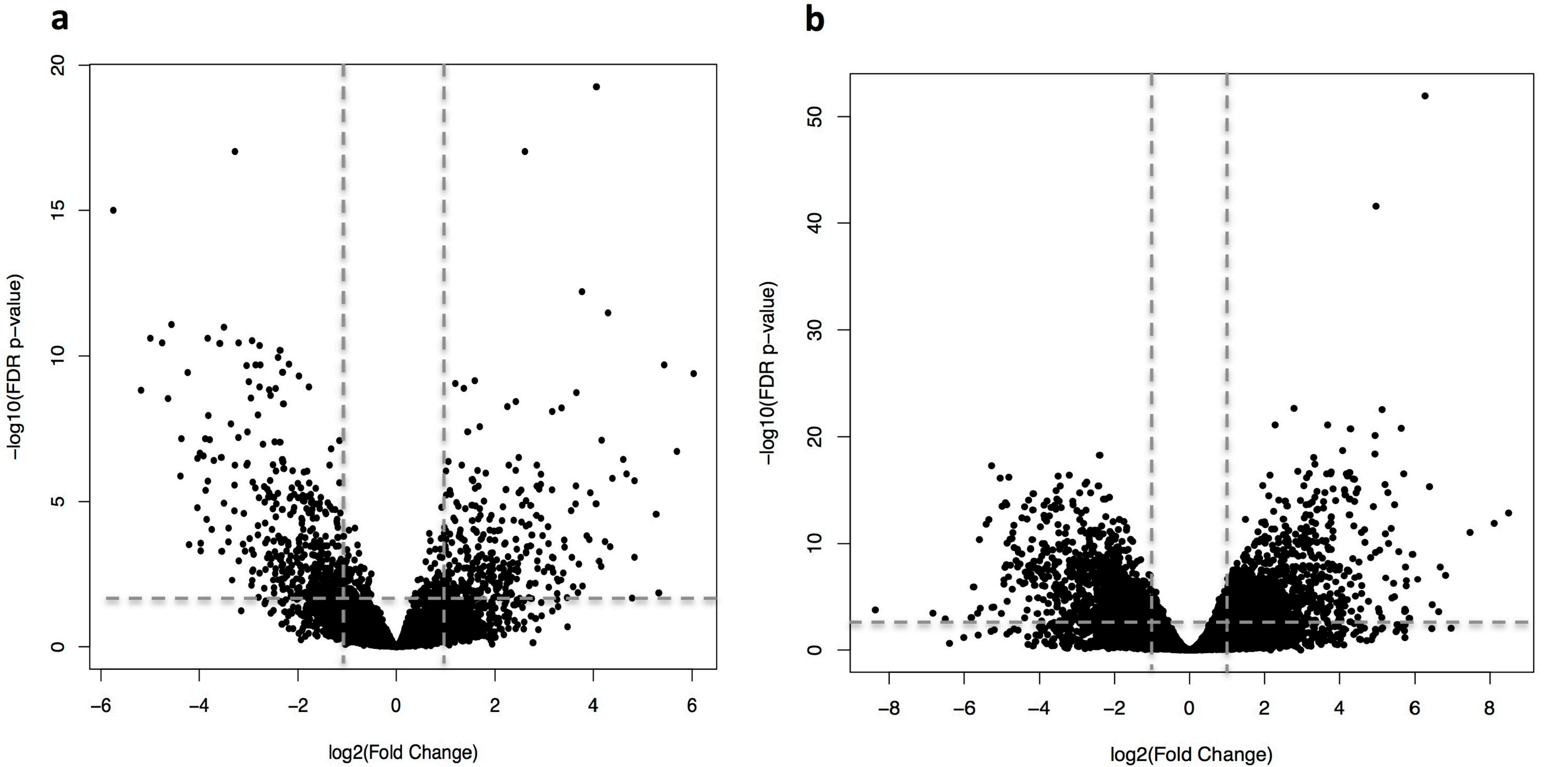
a
b
-log10(FDR p-value)
log2(Fold Change)
0
5
10
15
20
−6
−4
−2
0
2
4
6
0
10
20
30
40
50
−8
−6
−4
−2
0
2
4
6
8

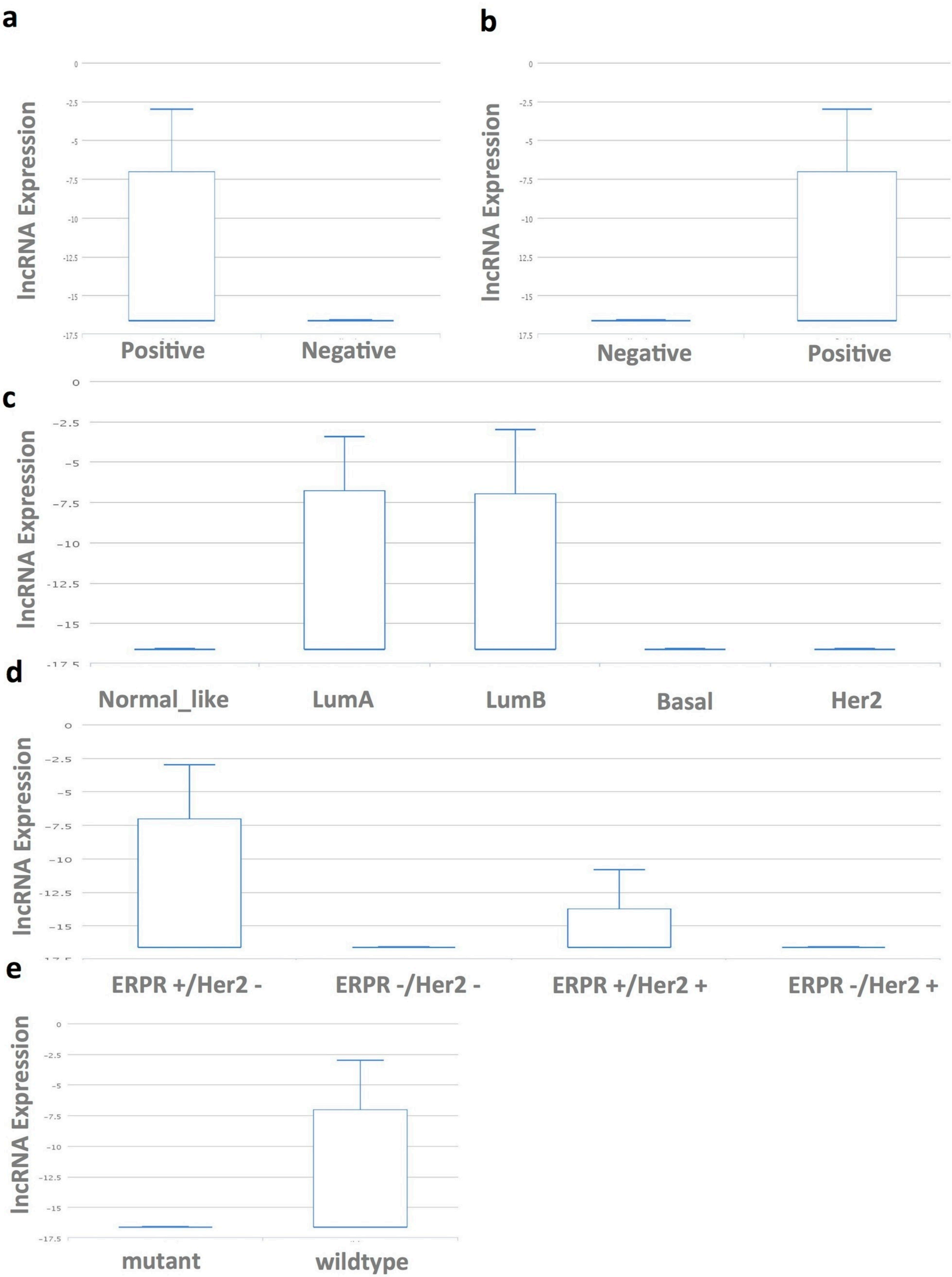
a
lncRNA Expression
0
-2.5
-5
-7.5
-10
-12.5
-15
-17.5
Positive
Negative
b
lncRNA Expression
0
-2.5
-5
-7.5
-10
12.5
-15
17.5
Negative
Positive
c
lncRNA Expression
0
-2.5
-5
-7.5
-10
-12.5
-15
-17.5
Normal_like
LumA
LumB
Basal
Her2
d
lncRNA Expression
0
-2.5
-5
-7.5
-10
-12.5
-15
ERPR +/Her2 -
ERPR -/Her2 -
ERPR +/Her2 +
ERPR -/Her2 +
e
lncRNA Expression
0
-2.5
-5
-7.5
-10
-12.5
-15
-17.5
mutant
wildtype

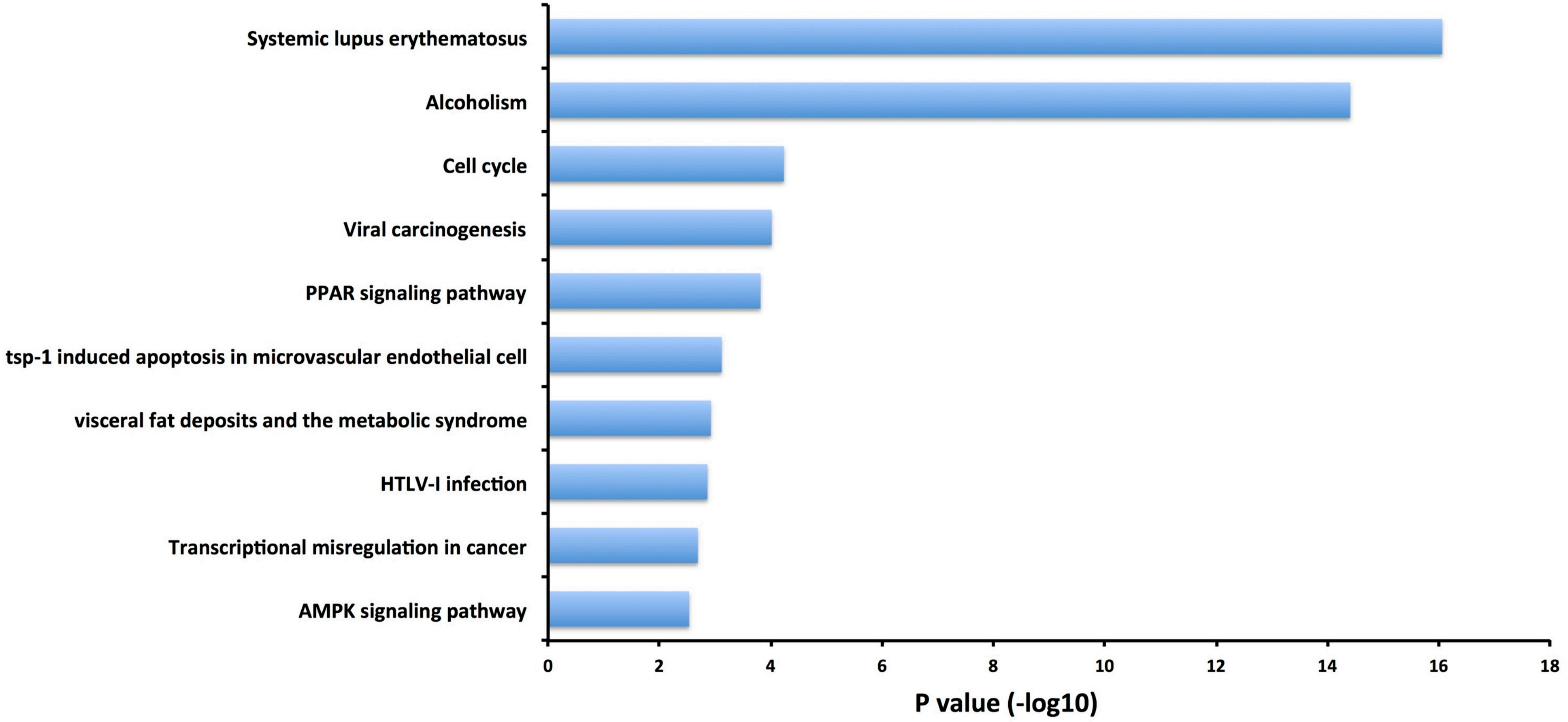

Systemic lupus erythematosus
Alcoholism
Cell cycle
Viral carcinogenesis
PPAR signaling pathway
tsp-1 induced apoptosis in microvascular endothelial cell
visceral fat deposits and the metabolic syndrome
HTLV-I infection
Transcriptional misregulation in cancer
AMPK signaling pathway
0
2
4
6
8
10
12
14
16
18
P value (-log10)

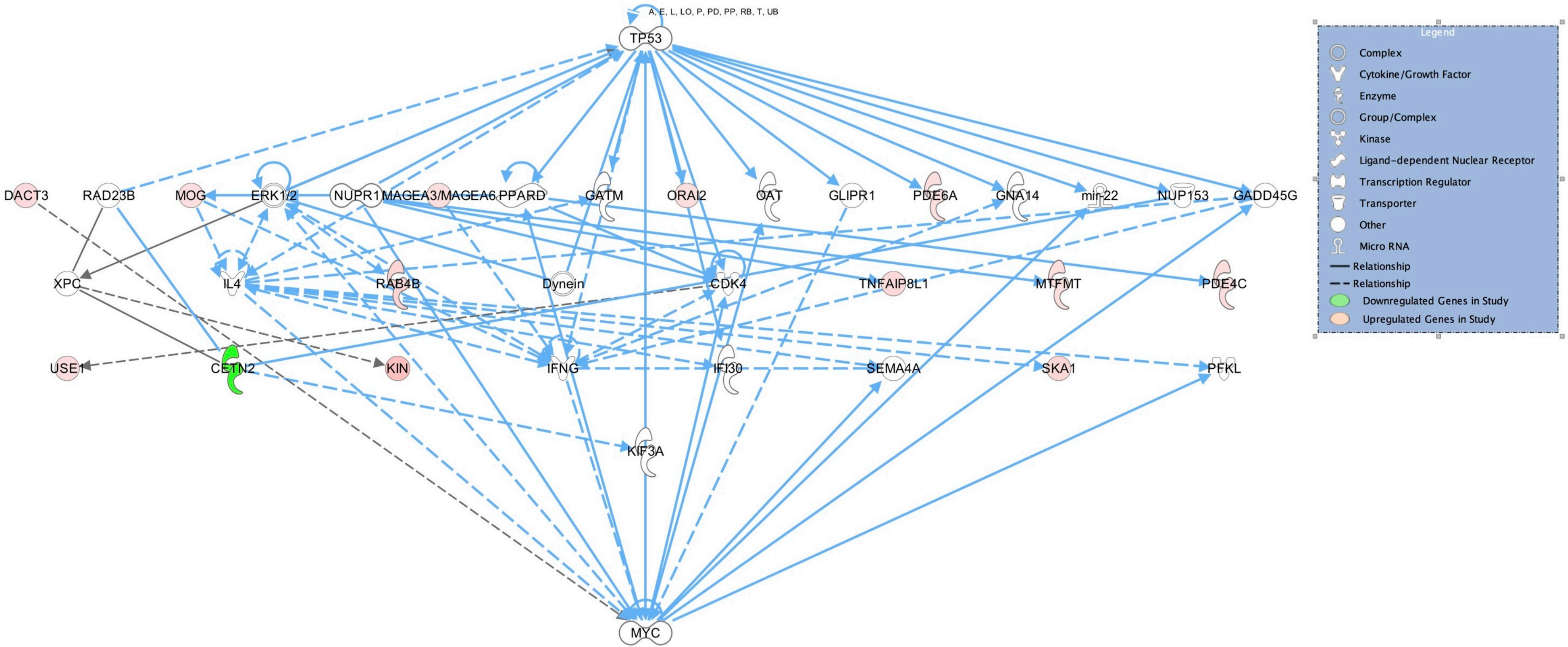
A, E, L, LO, P, PD, PP, RB, T, UB
TP53
DACT3
RAD23B
MOG
ERK1/2
NUPR1
MAGEA3/MAGEA6
PPARD
GATM
ORAI2
OAT
GLIPR1
PDE6A
GNA14
mir-22
NUP153
GADD45G
XPC
IL4
RAB4B
Dynein
CDK4
TNFAIP8L1
MTFMT
PDE4C
USE1
CETN2
KIN
IFNG
IFI30
SEMA4A
SKA1
PFKL
KIF3A
MYC
Legend
Complex
Cytokine/Growth Factor
Enzyme
Group/Complex
Kinase
Ligand-dependent Nuclear Receptor
Transcription Regulator
Transporter
Other
Micro RNA
Relationship
Relationship
Downregulated Genes in Study
Upregulated Genes in Study

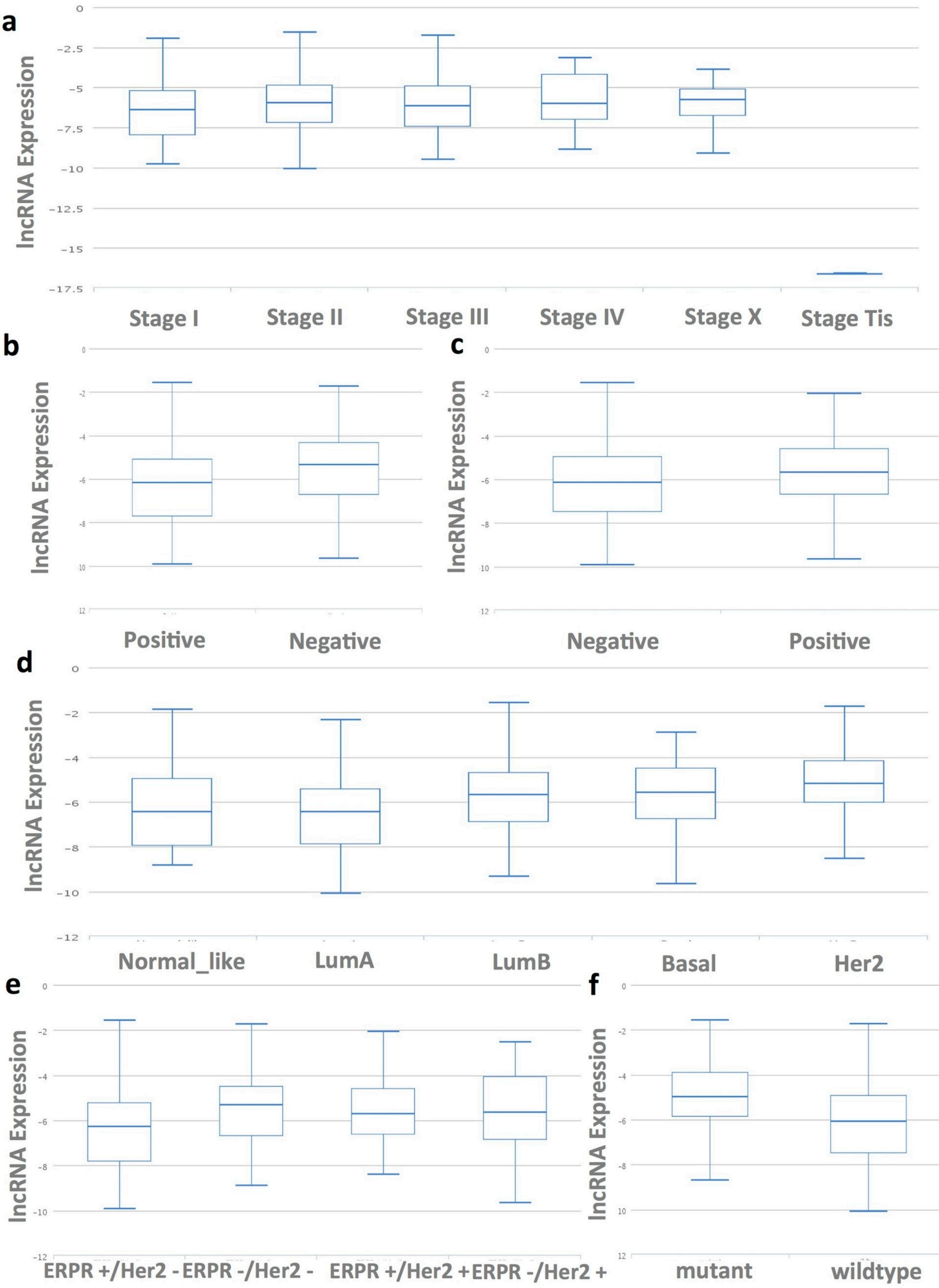

a
lncRNA Expression
0
-2.5
-5
-7.5
-10
-12.5
-15
-17.5
Stage I
Stage II
Stage III
Stage IV
Stage X
Stage Tis
b
lncRNA Expression
0
-2
-4
-6
-8
-10
-12
Positive
Negative
c
lncRNA Expression
0
-2
-4
-6
-8
-10
-12
Negative
Positive
d
lncRNA Expression
0
-2
-4
-6
-8
-10
-12
Normal_like
LumA
LumB
Basal
Her2
e
lncRNA Expression
0
-2
-4
-6
-8
-10
-12
ERPR +/Her2 -
ERPR -/Her2 -
ERPR +/Her2 +
ERPR -/Her2 +
f
lncRNA Expression
0
-2
-4
-6
-8
10
12
mutant
wildtype

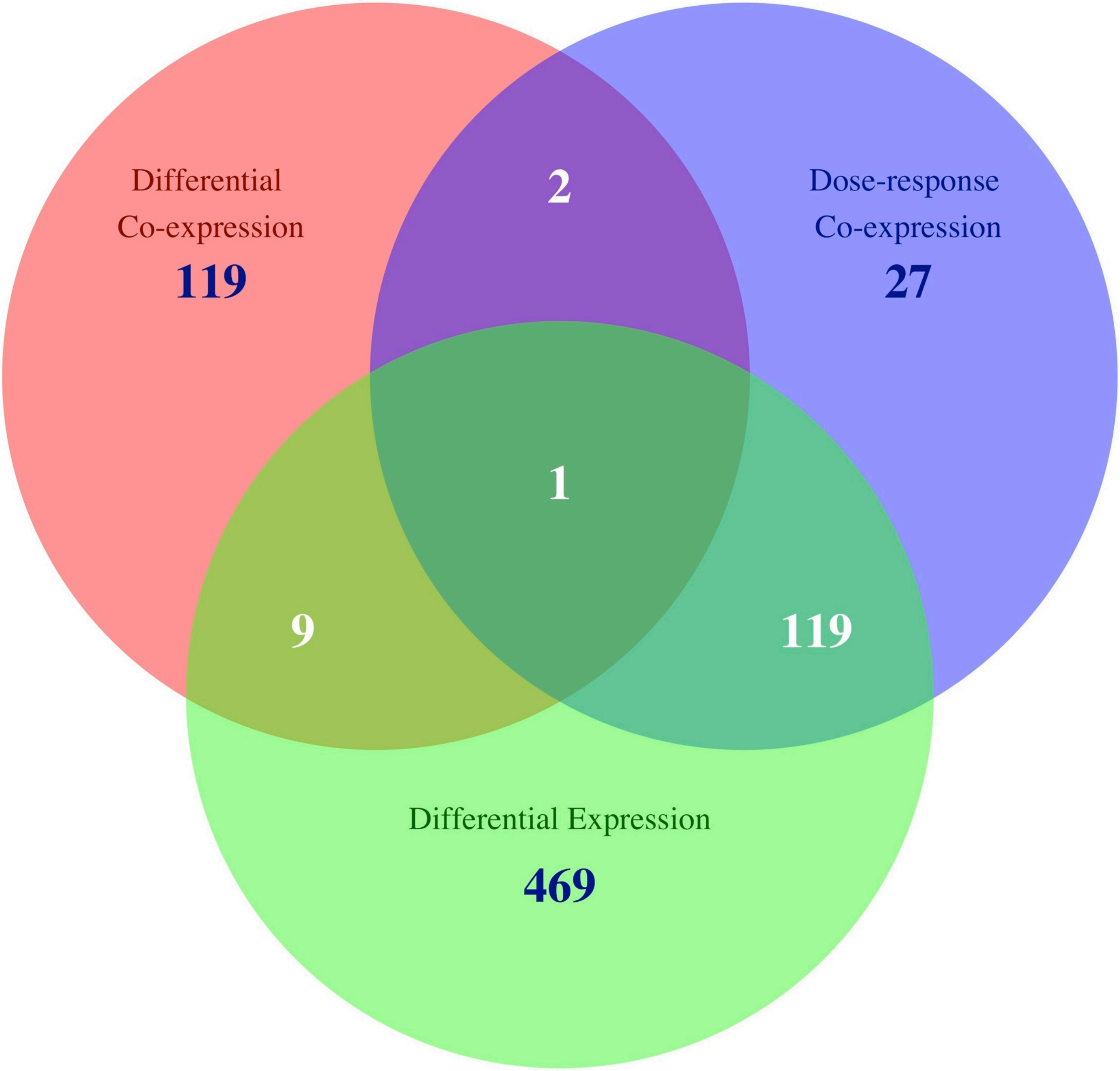
Differential
Co-expression
119
2
Dose-response
Co-expression
27
1
9
119
Differential Expression
469
